# Nitrogen use efficiency—a key to enhance crop productivity under a changing climate

**DOI:** 10.3389/fpls.2023.1121073

**Published:** 2023-04-18

**Authors:** Prabhu Govindasamy, Senthilkumar K. Muthusamy, Muthukumar Bagavathiannan, Jake Mowrer, Prasanth Tej Kumar Jagannadham, Aniruddha Maity, Hanamant M. Halli, Sujayananad G. K., Rajagopal Vadivel, Das T. K., Rishi Raj, Vijay Pooniya, Subhash Babu, Sanjay Singh Rathore, Muralikrishnan L., Gopal Tiwari

**Affiliations:** ^1^Division of Agronomy, Indian Council of Agricultural Research (ICAR)-Indian Agricultural Research Institute, New Delhi, India; ^2^Division of Crop Improvement, Indian Council of Agricultural Research (ICAR)-Central Tuber Crops Research Institute, Thiruvananthapuram, India; ^3^Department of Soil and Crop Sciences, Texas A&M University, College Station, TX, United States; ^4^Biotechnology Division, Indian Council of Agricultural Research (ICAR)-Central Citrus Research Institute, Nagpur, India; ^5^Crop, Soil and Environmental Sciences, Auburn University, Auburn, AL, United States; ^6^School of Soil Stress Management, Indian Council of Agricultural Research (ICAR)-National Institute of Abiotic Stress Management, Pune, India; ^7^Crop Protection, Indian Council of Agricultural Research (ICAR)-Indian Institute of Pulse Research, Kanpur, India; ^8^Division of Agricultural Extension, Indian Council of Agricultural Research (ICAR)-Indian Agricultural Research Institute, New Delhi, India

**Keywords:** conservation tillage system, NUE, nitrogen assimilation, nitrogen loss, QTLs

## Abstract

Nitrogen (N) is an essential element required for the growth and development of all plants. On a global scale, N is agriculture’s most widely used fertilizer nutrient. Studies have shown that crops use only 50% of the applied N effectively, while the rest is lost through various pathways to the surrounding environment. Furthermore, lost N negatively impacts the farmer’s return on investment and pollutes the water, soil, and air. Therefore, enhancing nitrogen use efficiency (NUE) is critical in crop improvement programs and agronomic management systems. The major processes responsible for low N use are the volatilization, surface runoff, leaching, and denitrification of N. Improving NUE through agronomic management practices and high-throughput technologies would reduce the need for intensive N application and minimize the negative impact of N on the environment. The harmonization of agronomic, genetic, and biotechnological tools will improve the efficiency of N assimilation in crops and align agricultural systems with global needs to protect environmental functions and resources. Therefore, this review summarizes the literature on nitrogen loss, factors affecting NUE, and agronomic and genetic approaches for improving NUE in various crops and proposes a pathway to bring together agronomic and environmental needs.

## 1 Introduction

A rapidly growing global population places considerable pressure on agricultural lands to produce more food and energy per unit area. For sustainable production, agricultural practices must both intensify productivity and simultaneously protect the environment and human and animal health. Improving nitrogen use efficiency (NUE) is an element of this framework (Zhang et al., 2015; Xiong et al., 2018). Nitrogen (N) is a key constituent of all living cells and is essential for the growth and development of plants. Fertilizer N is the second largest requirement after water in crop production, and N is the most common yield-limiting nutrient deficiency (Marschner, 1995). The ratio of N taken up *versus* the unit applied to a crop is referred to as NUE (Fageria and Baligar, 2005). The low N use of the crop indicates that uptake is inefficient or higher than the plant’s requirement (Anas et al., 2020). Cereal crops like rice, wheat, and maize require large amounts of N for healthy growth and higher yields (Linquist et al., 2012). Hence, varieties with higher NUE should be a priority for breeders developing new varieties (Balyan et al., 2016; Mălinaş et al., 2022).

The global estimates of N stored in soil are 65 Pg to 30 cm depth and 92–140 Pg to 100 cm depth (Zinke et al., 1986; Batjes, 2014). The largest portion of stores is in the form of organic N, which is not directly plant available. Chemical fertilizers and manures add 200 Tg of N each year (Potter et al., 2010). Biological N fixation provides an additional input of 258 Tg of N (Fowler et al., 2013). Ammonium (NH_4_^+^) and nitrate (NO_3_^−^) are the two forms of plant-available N. Globally, only 50% of applied N is converted and the rest is wasted (Mălinaş et al., 2022).

Crop NUE is influenced by environmental factors, plants’ physiological activity, and their interactions. Biochemical transformations of N in soil are complex and are best considered as being in a state of continual flux (**Table 1**). The fluxes of biochemical transformation in the soil system are primarily responsible for constraints to NUE. However, physical losses of N from the plant or soil system also decrease NUE. The major forms of N loss are the volatilization of ammonia (NH_3_) gas, leaching of dissolved NO_3_^−^, and overland runoff of all soluble forms. Changes in temperature and precipitation patterns affect biological and enzyme activity rates, which are important for most transformations listed in **Table 1**.

**Table 1:** Nitrogen transformation processes.

Nitrogen transformation process	Chemical equation	Eq.	The direction of the PAN flux
Biological fixation (enzymatic fixation of atmospheric N_2_ to NH_3_)	$N_{2}+8H^{+}\to 2NH_{3}+H_{2}$	10	Input
Plant uptake of N as NH_4_^+^	$\left(Plant\right)ROH+NH_{4}^{+}↔RNH_{2}+H_{2}O+H^{+}$	11	Neutral (*if target crop*) Loss (*if non-target*)
Plant uptake of N as NO_3_^−^	$\left(Plant\right)ROH+NO_{3}^{-+}H^{+}+2CH_{2}O↔RNH_{2}+2CO_{2}+2H_{2}O$	12	Neutral (*if target crop*) Loss (*if non-target*)
Urea hydrolysis (enzymatic hydrolysis of urea)	${\left(NH_{2}\right)}_{2}CO+H_{2}O+H^{+}↔2NH_{4}^{+}+CO_{2}$	13	Input
Nitrification (enzymatic oxidation of ammonium to nitrate)	$NH_{3}+2O_{2}↔NO_{3}^{-}+2H^{+}+H_{2}O$	14	Neutral
Denitrification (anaerobic enzymatic reduction of NO_3_^−^ to N_2_ gas)	$5CH_{2}O+4NO_{3}^{-}+4H^{+}↔2N_{2}+5CO_{2}+3H_{2}O$	15	Loss
Volatilization of N as NH_3_	$NH_{4}^{+}\;\overset{\;}{↔}\;NH_{3}^{0}+H^{+}\;\left(pKa=9.3\right)$	16	Loss
Ammonification (enzymatic mineralization of organic N)	$RNH_{2}+H_{2}O+H^{+}↔ROH+NH_{4}^{+}$	17	Input
Immobilization (uptake and incorporation into microbial biomass)	$NH_{4}^{+}+ROH↔RNH_{2}+H_{2}O+H^{+}$	18	Loss

PAN, plant-available nitrogen.

## 2 Approaches to evaluating NUE

The simplest approach to quantifying NUE is to divide the crop yield (*Y*) by the nitrogen inputs (*N*) (Eq. 1).


(1) NUE =Y÷N 

However, several authors have suggested that yield may be defined in several ways, including the mass of the harvested portion of the crop, total (aboveground) biomass of the crop, N content contained in the harvestable portion, and N content of the total biomass. Fageria and Baligar (2005) proposed a number of general “groups” of approaches to calculate NUE that may be considered (Eqs. 2-7).


(2) $$\text{Agronimic}\;\text{Efficiency}\;(\text{AE})=\left(\frac{{\text{G}}_{\text{f}}-{\text{G}}_{\text{u}}}{{\text{N}}_{\text{a}}}\right)$$

Where *G*_f_ and *G*_u_ are the grain yields (kg) of the fertilized and unfertilized plots, respectively, and *N*_a_ is the rate of N applied (kg).


(3) $$\text{Physiological}\;\text{Efficiency}\;(\text{PE})=\left(\frac{(\left({\text{Y}}_{\text{f}}-{\text{Y}}_{\text{u}}\right)}{\left({\text{N}}_{\text{f}})-({\text{N}}_{\text{u}}\right))}\right)$$

Where *Y*_f_ and *Y*_u_ are the total aboveground biomass (kg) of the crop in fertilized and unfertilized plots, respectively, and *N*_f_ and *N*_u_ are the N contents (kg) of the aboveground biomass in the fertilized and unfertilized plots, respectively.


(4) $$\text{Agrophysiological}\;\text{Efficiency}\;(\text{APE})=\left(\frac{(\left({\text{G}}_{\text{f}}-{\text{G}}_{\text{u}}\right)}{\left({\text{N}}_{\text{f}})-({\text{N}}_{\text{u}}\right))}\right)$$

Where *G*_f_ and *G*_u_ are the grain yield in fertilized and unfertilized plots, respectively.


(5) $$\text{Apparent Recovery}\;\text{Efficiency}\;(\text{ARE})=\left(\frac{{\text{N}}_{\text{f}}-{\text{N}}_{\text{u}}}{{\text{N}}_{\text{a}}}\right)$$

(6) $$\text{Utilization}\;\text{Efficiency}\;(\text{UE})=\left(\frac{{\text{Y}}_{\text{f}}-{\text{Y}}_{\text{u}}}{{\text{N}}_{\text{a}}}\right)$$

All of the above equations rely on the assumption that varied nitrogen rate as fertilizer input is the independent variable. Naturally, as the mass of N inputs decreases, the calculated efficiency increases in equations using N rate or difference in N accumulation in the denominator. It would therefore be quite easy to interpret these as suggesting that the lowest rates of N fertilizer inputs result in the best NUE. This outcome ignores the importance of crop productivity.

Berendse and Aerts (1987) proposed a “biologically meaningful” definition of NUE as the product of nitrogen productivity (*An*/*L*_n_) and the mean residence time (1/*L*_n_) of nitrogen in the plant (Eq. 7).


(7) $$\text{Biologically Meaningful}\;\;\text{NUE}=\left(\frac{{\text{A}}_{\text{n}}}{{\text{L}}_{\text{n}}}\right)$$

This approach avoids the same pitfalls in Eqs. 1-6 but somehow fails to provide an interpretation of NUE necessary to evaluate the direct effects of climate change or advancements in crop management to adapt to climate change. It is indeed likely that future studies will not employ varied rates of N inputs to study NUE but will instead evaluate changes in other practices, varieties, genetic enhancements, and emerging biotechnologies. In this case, new approaches to the calculation of NUE will be needed. Preferably, these will also include mass balances of native soil plant-available N (PAN) and potential PAN in addition to fertilizer or manure inputs.

When considering the pressures of climate change, increased atmospheric carbon dioxide (CO_2_) will impact the ultimate equilibrium states of many of these processes. Higher temperatures will reduce soil N inventories by 5%–10% due to increased mineralization (Fowler et al., 2015). With the twin pressures of population expansion and climate change, management and breeding will need to focus on fundamental problems to make progress in NUE. Consider, for example, that leaf expansion and photosynthetic rates are affected by low N and that root traits are chiefly responsible for N uptake and NUE in maize (Wijewardana et al., 2015). Inbred maize lines exhibiting higher NUE were those with larger root diameters (Wijewardana et al., 2015). *Root-ABA1*, a major quantitative trait locus (QTL) for root development in maize, plays a vital role in NUE along with four other QTLs, viz., *Qaer3.10*, *Qaer5.05–6*, *aer9.07–8*, and *Qaer10.04*, responsible for aerenchyma cell development. In rice, the transcriptomic approach has helped to identify 62 candidate NUE genes. *SHORT ROOT* and *SCARECROW* are root-patterning genes responsible for root development and architecture. AUX1 and PIN proteins regulate the auxin movement and lead to lateral root development. NUE is a complex trait governed by the crop’s agronomic, physiological, environmental, and genetic traits. The integration of association mapping and genomics approach accompanied by the phenomic approach will be a major contributor to improve the NUE of global crops (Wani et al., 2021). Therefore, it is increasingly important to improve our understanding of factors affecting NUE and possible management measures for improving the NUE of crops.

This review focuses on describing different forms of N loss in the environment, analyzing the factors influencing NUE, discussing the consequences of poor NUE, and suggesting possible management practices for enhancing the NUE in various crops. Overall, better agronomic management of crops, genetic resources, breeding programs, and biotechnological tools to improve NUE are presented as potential solutions to low NUE of crops.

## 3 Loss of N in the soil environment

### 3.1 N loss pathways

The negative effect of N loss on water, the environment, and human and animal health has been well reported (Singh et al., 2010). Soil N is transient and moves rapidly away from the point of application through various mechanisms. The processes responsible for N loss include volatilization, nitrification, denitrification, leaching, surface runoff, ammonium fixation, and immobilization (Baggs et al., 2000). Overall, the amount of mineral N in the soil at any given time can be described by the following N balance equation (Eq. 19) (Di and Cameron, 2002**)**.


(19) N=Np+Nb+Nf+Nu+Nm−Npl−Ng−−Nl−Ne

where *N*_p_ is the precipitation and dry deposition, *N*_b_ is the biological fixation (Eq. 10, **Table 1**), *N*_f_ is the fertilizer, *N*_u_ is the urine and dung return to the soil, *N*_m_ is the mineralization, *N*_pl_ is the plant uptake (Eqs. 11 and 12, **Table 1**), *N*_g_ is the gaseous losses, *N*_i_ is the immobilization, *N*_l_ is the leaching loss, and *N*_e_ is the erosion and surface runoff.

#### 3.1.1 Volatilization

The gaseous loss of NH_3_ is known as volatilization. Volatilization is a complex process that is controlled by the physical, chemical, and biological properties of soil and the environment (Fan et al., 2011). Agriculture activities account for 50% of the total annual global NH_3_ loss (32 Tg year^−1^) to the atmosphere through volatilization (Liu et al., 2019a). Fertilizer and manure application and livestock activity are the primary sources of NH_3_ emissions in agriculture. Chemical N fertilizer alone is responsible for 34% of the loss (He et al., 2014). In particular, urea-based fertilizers are more susceptible than other N fertilizers because of the temporary increase in soil pH through the consumption of H^+^ ions during hydrolysis (Eq. 13). There is an equilibrium between NH_4_ and NH_3_ in soil solution (Eq. 16). The p*K*_a_ for equilibrium in Eq. 16 is 9.3. Therefore, alkaline conditions favor greater proportions of NH_3_ (Havlin et al., 2014). When soil pH exceeds 7.5, temperatures increase up to 45°C, sufficient air movement is present to remove NH_3_ gas at the soil–atmosphere interface, and losses of N as NH_3_ are maximized (Bock and Kissel, 1988; Havlin et al., 2014). Application to acidic soils raises a little risk of volatilization. Application to sandy soils with low native cation exchange capacity (CEC) raises the risk. The common management approaches to improve the NUE of NH_4_/NH_3_ fertilizers include incorporation into the soil through injection or tillage to protect NH_4_/NH_3_ through the association of NH_4_ with clay colloid cation exchange sites. When animal wastes are used as nutrient sources for crops, volatilization has been markedly diminished by incorporation or pretreatment with acidifying agents (Marshall et al., 1998; Choi and Moore, 2008; Doydora et al., 2011). Splitting applications between pre-plant and one or more subsequent applications later in the growing season is also commonly recommended to reduce the time that NH_4_/NH_3_ fertilizers are exposed to environmental conditions that promote loss.

#### 3.1.2 Urea hydrolysis

Urea hydrolysis (Eq. 13) may be considered the final step in the mineralization of organic N. The urease enzymes (urea amidohydrolases, EC 3.5.1.5) are produced by a large number of organisms filling a variety of ecological niches including plants, bacteria, algae, fungi, and invertebrates (Sigurdarson et al., 2018). In most soils, the enzyme is more than sufficiently present and free in solution to rapidly hydrolyze urea to NH_3_ (Klose and Tabatabai, 1999). Therefore, management to avoid losses of NH_3_ through volatilization following urea application has commonly involved the inhibition of ureases to prevent the reaction from occurring until the urea itself may be safely incorporated into the subsurface soil.

Conventional urease inhibitors include N-(n-butyl) thiophosphoric triamide (NBPT), perhaps the most widely employed, with demonstrated effectiveness in rice, cotton, wheat, maize, and pasture grasses (Zaman et al., 2009; Kawakami et al., 2012; Marshall et al, 1998; Martins et al., 2017; Wallace et al., 2020). Urease inhibition with NBPT and cyclohexylphopshoric triamide (CHPT) may also be effective in preventing N losses from manure sources (Svane et al., 2020). Plant-based materials such as those isolated from *Canavalia ensiformis* (jack bean), *Eucalyptus camaldulensis* (eucalyptus), allicin from *Allium sativum* (garlic), and certain *Acacia* spp. have been shown to inhibit ureases in soil (Mathialagan et al., 2017; Rana et al., 2021). This raises the possibility of the increased entrance of plant biotechnologies into this area. Finally, as with any N source, urea may also be split applied and/or subsurface applied to prevent exposure to environmental conditions that lead to losses.

#### 3.1.3 Leaching

Higher rates of animal manure or commercial N fertilizer application increase NO_3_^−^ leaching as a result of increased available N concentration in soil solution. Nitrate N is highly susceptible to leaching due to the negative charge associated with NO_3_^−^ which prevents its association with negatively charged soil colloids, whereas NH_4_^+^ is electrostatically attracted to colloids and therefore protected from leaching (Lodhi et al., 2009). Therefore, rain and irrigation would take the NO_3_^−^ out of the system. Nitrate leaching takes place mainly after the heavy rainy season and the period of slow crop growth. Pande et al. (1985) reported that the N leaching process accounted for 2%–60% of the applied N loss. It has been estimated that the irrigated wheat fields account for 5 to 12.5 kg N ha^−1^ N leaching loss, where farmers have applied 250 kg N ha^−1^ with two splits in northern Mexico (Riley et al., 2001). Clay soil typically has lower NO_3_^−^ leaching than sandy soil due to limited hydraulic conductivity. In clay soils, NO_3_^−^ measured in soil samples to 60 cm can be subtracted from maize N fertilizer recommendations due to the reduced leaching potential (Fromme et al., 2017).

#### 3.1.4 Nitrification

Nitrification is a microbial process (Eq. 14, **Table 1**), in which the ammonium is converted into nitrate by the oxidation process (Ward et al., 2011). It is a two-stage process (Eqs. 8 and 9) and is mediated by autotrophic bacteria.


(8) NH3+112O2→NO2−+H2O+H++84 kcal mol−1

(9) NO2+112O2→NO3−+17.8 kcal mol−1

The first stage is initiated by the ammonia-oxidizing bacteria like *Nitrosospira* and *Nitrosomonas*, which perform the oxidation of NH_4_^+^ to nitrite (NO_2_^−^) by means of the membrane-bound ammonia monooxygenase enzyme associated with hydroxylamine oxygenase (Jiang et al, 2018). The second step involves the conversion of NO_2_^−^ to NO_3_^−^ mediated by *Nitrobacter*. The last stage is much faster and more effective than the first stage; hence, nitrite rarely accumulates in the soil (Linn and Doran, 1984). Nitrification takes place in an aerobic soil environment with optimal soil moisture (60% water-filled pore space) (Linn and Doran, 1984). However, it is a very slow process in anaerobic soil environments (rice ecosystem) (Linn and Doran, 1984). The process is also regulated by soil temperature, pH, NH_4_^+^/NH_3_ concentration, and microbial population (Sharma and Ahlert, 1977). Nitrate produced by this process can be leached, absorbed by plants, and immobilized by soil microorganisms.

#### 3.1.5 Denitrification

**Figure 1:**
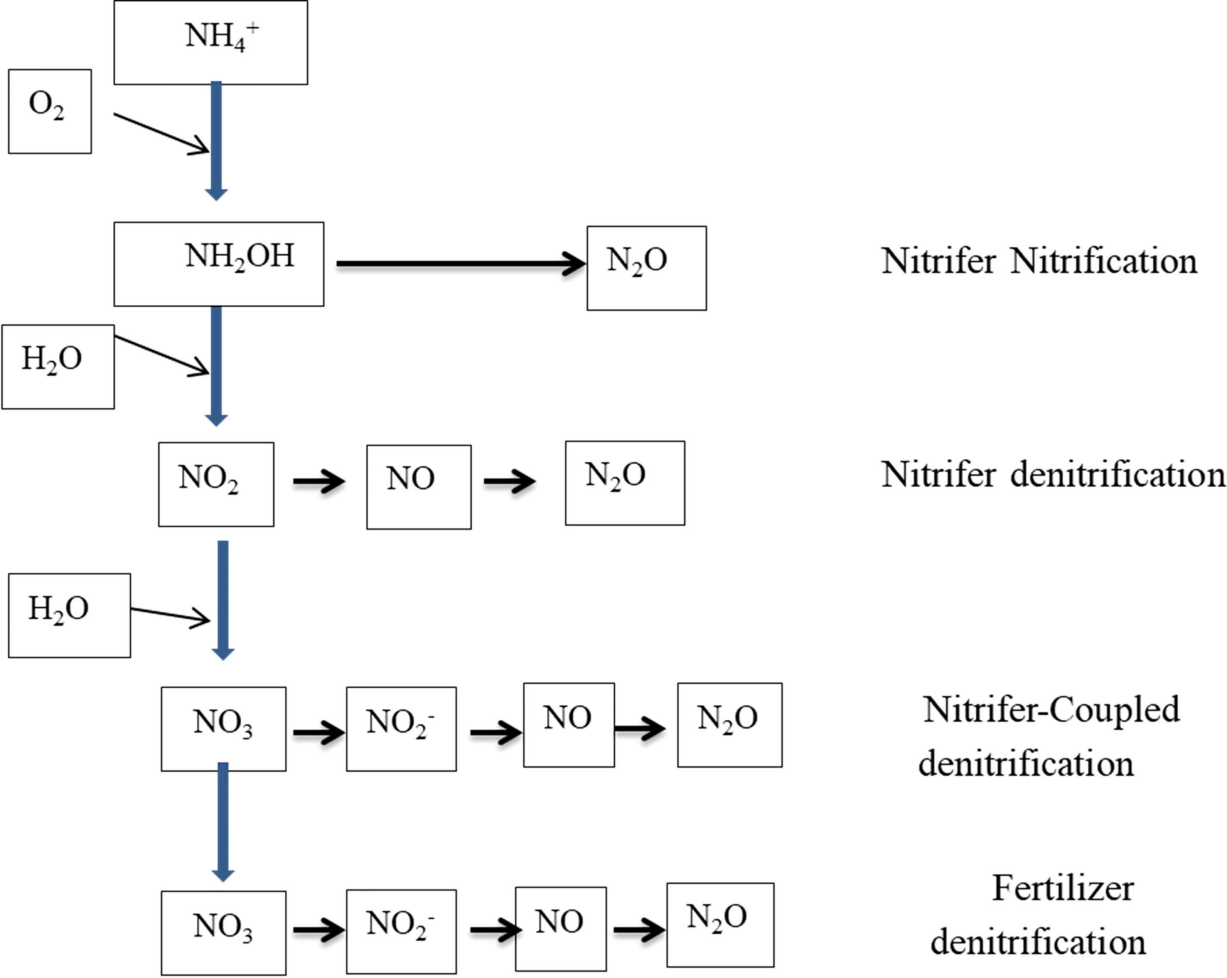
Different pathways of N_2_O production in soil (Kool et al., 2011).

Denitrification is also a microbe-mediated, though strictly anaerobic, process (Eq. 15, **Table 1**) wherein NO_2_^−^ is reduced to N_2_ gas using intermediate products such as nitrogen dioxide [NO_2_, nitric oxide (NO), and nitrous oxide (N_2_O) (**Figure 1**)]. The production of N_2_O is a major concern because of its greenhouse gas (GHG) function, with approximately 300 times the GHG potential of CO_2_. Soil N loss through denitrification as a percentage of applied N varies widely and is a function of soil water content, soluble carbon (C), the presence of NO_3_^−^, temperature, and time. Global loss of N from denitrification is estimated to be 96 Tg year^−1^ in 2000 and would probably increase to 142 Tg year^−1^ by 2050 (Bouwman et al., 2013). The process is carried out by a group of facultative anaerobic bacteria and catalyzed by nitrate reductase and nitrite reductase enzymes (Garbeva et al., 2007; Ranatunga et al., 2018). Two different electron acceptors are used during the denitrification process in aerobic conditions; oxygen acts as an electron acceptor, while NO_3_^−^ is used as an electron acceptor in anaerobic conditions (Bock et al., 1995). Chemo-denitrification is another process responsible for nitrous oxide emission, but the quantity is smaller than biological production (Kool et al., 2011). Likewise, the nitrification process also releases N_2_O through the spontaneous oxidation of hydroxylamine, which is an intermediate in the nitrification process (Kool et al., 2011).

Conventional management for the prevention of denitrification losses has conventionally been through inhibition of nitrification in soil. Nitrification inhibition prevents the formation of NO_3_^−^, the substrate for denitrification, from NH_4_^+^ (Eq. 14). There are a number of chemistries known and used in agriculture for nitrification inhibition. These include nitrapyrin (and various other pyridines), thiourea, thiophosphoryl triamide (also a urease inhibitor), 3,4-dimethylpyrazole phosphate (DMPP), and dicyandiamide (DCD) (McGinn et al, 2016; Ruser and Schulz, 2015; Alonso-Ayuso et al., 2016). Each of these chemistries is known to increase the production and release of nitrous oxide (N_2_O) from soils, a fact that should be considered in all efforts to increase NUE.

Research into biological nitrification inhibition (BNI) is advancing rapidly (Coskun et al., 2017). The current state of BNI research suggests that both plant-derived compounds (direct inhibition) and indirect mechanisms may be simultaneously responsible. For a thorough review of isolated plant exudates and metabolomics responsible for BNI, please see Nardi et al. (2020). Harnessing BNI for agricultural scale use will continue to be a fecund area of research in the near future for plant biotechnology and breeding disciplines.

#### 3.1.6 Soil erosion and runoff

Slope, rainfall intensity, soil type, and vegetation are key determinants of soil and nutrient loss and transport (Kang et al., 2001). Soil nearest to the surface often contains the greatest concentrations of N and organic matter which can be readily transported through runoff and erosion. It is possible that up to 70% of surface-applied N fertilizer may be lost to a runoff if rain occurs on the same day (Mandal et al., 2012).

Management of cropping systems to reduce such physical losses of N will improve NUE. Conventional approaches to minimizing erosion and runoff include reduced tillage or no-tillage, cover cropping, surface residue retention (conservation tillage), contour tillage, terracing, and grassed waterways (Boincean and Dent, 2019; Farzadfar et al., 2021; Young et al., 2021). While no-till and reduced till systems tend to protect or increase soil organic matter, which includes organic nitrogen, Canisares et al. (2021) reported that no-till increased mineralization rates without affecting the optimal corn fertilization response. In this case, yields were greater under no-till (~1,000 kg ha^−1^), though the response to N fertilizer was unchanged. Depending on how it is defined (Eqs. 1-7), NUE may or may not have been improved in this case. However, efforts to control erosion and loss of N through reduced tillage should improve soil stocks of N through both conservation and enhanced mineralization and continue to be the best recommended practices.

When cover crops are included in cropping systems, there are multiple mechanisms that can lead to increased NUE. Reduction of erosion caused by overland flow is more effective when covers with finer roots such as cereal rye or oats are used as opposed to covers with thick roots such as mustards or radishes (De Baets et al., 2011). Leguminous cover crops fix N_2_ gas from the atmosphere into plant-available NH_3_ (Eq. 10) and incorporated into the plant biomass. Upon senescence of the cover crop, the biomass N may then be remineralized (Eq. 18). Any measure of NUE which simply considers the reduction of fertilizer requirement will naturally be improved by increasing soil stores in this way. Cereal covers have the potential to reduce leaching by scavenging N from soils into biomass and releasing to the following cash crop through mineralization as well. Ranells and Wagger (1997) reported that the legumes hairy vetch and crimson clover could release 132 and 60 kg N ha^−1^, respectively, while the non-legume cereal rye released 24 kg ha^−1^.

#### 3.1.7 Interlayer fixation of NH_4_^+^ by clay minerals

Ammonium fixation occurs with 2:1 type of clay minerals such as illite, vermiculite, and smectite because they have negative charges and have the ability to expand interlayer spacing when soil water enters the basal oxygen plane (Nieder et al., 2011). The NH_4_^+^ ion is comparable to that of K^+^ with respect to ionic radii and low energy of hydration (Nieder et al., 2011). Therefore, the NH_4_^+^ ion is fitted exactly in the ditrigonal holes, or interlayers, in the basal oxygen plane of 2:1 clay mineral when soil water is present (Kunze and Jeffries, 1953). The clay mineral interlayers collapse approximately 1 nm upon drying, and NH_4_^+^ ions are then trapped between silicate sheets and largely removed from further exchange reactions (Juang et al., 2001).

#### 3.1.8 Immobilization of N in soils

**Figure 2:**
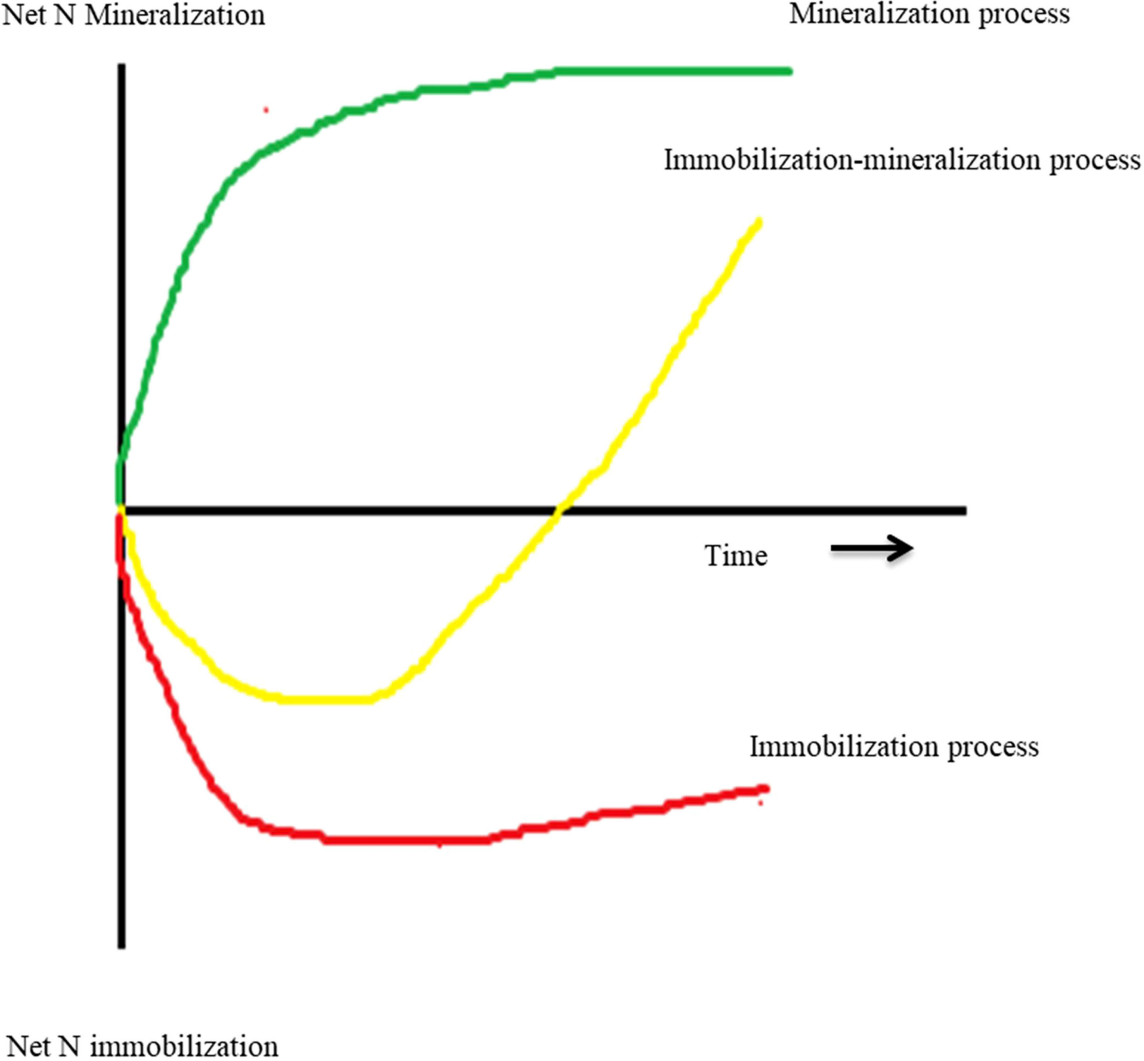
Three different process types regarding the effects of returning plant residues on soil inorganic N over the limited experimental period (Chen et al., 2014).

Manure and residues are applied to the soil as a source of nutrients (**Figure 2**). The first step after applying organic matter to the soil is mineralization (Eq. 17, **Table 1**), which converts the unavailable nutrient form into the available form NH_4_^+^ (Chen et al., 2014). The C:N ratio of organic matter influences the N mineralization process because microbial biomass production requires both N and C (Chen et al., 2014). The wider the C:N ratio (e.g., >30:1) could hinder the mineralization process due to insufficient N content, and this condition leads to the immobilization of N (Eq. 18) (Quemada and Cabrera, 1995; Yassen et al, 2010). Immobilization is a process by which applied N can be incorporated into microbial biomass to provide for protein synthesis and reproduction. When mineral N + mineralizable organic N are insufficiently present to meet these needs, immobilization will remove plant-available N from the system (Sakala et al., 2000; Bird et al., 2001). Immobilization is considered negligible when the C:N ratio is <20:1.

In addition, when the N concentration is insufficient at the early stage of residue decomposition, the N content of the microbe’s own tissues may be increased through the remineralization process (Zelenev et al., 2006). Remineralization is a natural process by which the microbes requiring N can meet by mineralization of dead microorganisms using the enzymolysis process. Shindo and Nishio (2005) reported that the remineralization rates of wheat straw were 0.71, 0.55, and 0.29 mg N kg^−1^ day^−1^ after 7, 28, and 54 days, respectively. The high rate of remineralization is usually happening due to high consumption and low assimilation of N by microbes (Braun et al., 2018).

## 4 Factors affecting agronomic NUE of various crops

The agronomic N use efficiency of crops is greatly influenced by crop characteristics, environmental variability, and management practices.

### 4.1 Crop factors

Crops and crop varieties differ considerably in their ability to uptake N per unit of biomass production. The agronomic NUE of major crops is given in **Table 2**. Crops grown in well-irrigated conditions have a greater agronomic NUE than in unirrigated/rainfed conditions. A study conducted on various irrigation regimes on wheat in China concluded that the nitrogen partial factor productivity was higher for 40 mm per irrigation (41.57 to 43.69 kg grain per kg N applied) compared with 20 mm per irrigation (32.24 to 32.47 kg grain per kg N applied) (Si et al., 2020). High crop growth rate, yield, and N uptake in crops can be achieved by maintaining optimal soil moisture conditions (Giller et al., 2004; Ding et al., 2021). Annual crops have a higher agronomic NUE than perennial crops due to the higher N uptake efficiency and N concentration (Weih et al., 2011). However, yield-specific N efficiency was more for perennial crops than wheat (Weih et al., 2011). Compared with food crops, fodder crops have a higher agronomic NUE because of the higher biomass production per unit area and time.

**Table 2:** Agronomic NUE of various field crops in the world.

Crops	Agronomic NUE (kg grain kg^−1^ of applied N)	Country	References
Rice			
Irrigated	23	Brazil	Fageria and Baligar (2005)
Rainfed	21.18	Brazil	Fageria et al. (2014)
Wheat			
Irrigated	22-26	Nepal and Afghanistan	Fazily et al. (2020); Rawal et al. (2022)
Rainfed	22.9-23	Spain and Mexico	López-Bellido et al. (2005); Limon-Ortega (2021)
Corn			
Irrigated	14-27	India	Wang et al. (2014); Davies et al. (2020)
Rainfed	18-20	India	Sravanthi et al. (2017)
Mustard	13-21	India	Keerthi et al. (2017)
Sugarcane	230-241		Ghaffar et al. (2012)
Cotton	5 kg lint		Snider et al. (2021)
Fodder pearl millet	632 kg	India	Shekara et al. (2020)

### 4.2 Environmental factors

Important environmental factors that affect the agronomic NUE are photosynthetic active radiation (PAR), temperature, and rainfall. Environmental factors that affect the agronomic NUE of crops in decreasing order are temperature > rainfall > irradiance (Balasubramanian et al., 2004). The temperature requirement of crops may vary greatly (**Table 3**). For crops like rice and wheat, NUE increased significantly with increasing growing season temperature, but it decreased for corn, which may be due to the variation in plant N demand and uptake responses to temperature (Yu et al., 2022). An et al. (2005) reported that when the crop suffers because of lower than optimal temperature, an increase in seasonal air temperature suddenly increases crop growth and nitrogen demand, which could increase NUE. At low temperatures, the ability to absorb N by the roots is greatly reduced due to the high affinity of the temperature and nitrate influx systems in the roots (Glass, 2003). However, the increase in temperature may lead to a high loss of N, thus reducing the NUE (Bai et al., 2013). The N loss and crop N uptake are highly influenced by the intensity, duration, and frequency of rainfall in a crop season. The occurrence of rainfall within a day of N fertilizer application had a positive impact on the NUE. A strong correlation between the total rainfall and NUE was observed for the dryland summer sorghum in Australia (Rowlings et al., 2022). The highest NUE was reported for 125% simulated rainfall for wheat and corn in a silt loam soil of Kentucky, USA (Shahadha et al., 2021). Photosynthetic active radiation is a major driving force affecting crop growth and N uptake (Shahadha et al., 2021). However, it is only important for tropical and subtropical regions but not for temperate regions (Balasubramanian et al., 2004). Studies have observed that crop growth and nitrogen uptake vary significantly during the dry and wet seasons, mainly due to variations in PAR in the tropics (Balasubramanian et al., 2004).

**Table 3:** Temperature and water requirement of major crops.

Crops	Temperature (°C)	Water (mm)	References
Rice	25–35	900–2,500	Ruser and Schulz, 2015; Nishad et al. (2018)
Wheat	16–23	450–650	Ruser and Schulz, 2015; Khan et al. (2020)
Corn	25–33	500–800	Ruser and Schulz, 2015; Wild et al, 2001
Sugarcane	21–27	1,500–2,500	Ebrahim et al. (1998); Ruser and Schulz, 2015
Cotton	25–45	700–1,300	Ruser and Schulz, 2015; Shahadha et al, 2021
Chickpea	10–30	250–300	Ruser and Schulz, 2015; Devasirvatham et al. (2012)
Groundnut	20–30	500–700	Rana et al, 2021; Ruser and Schulz, 2015
Sunflower	25–28	250–350	Ruser and Schulz, 2015; Guo et al. (2021)

### 4.3 Management factors

Globally, 50% of the nitrogen applied to crops is lost to the environment, resulting in resource wastage and increased GHG emissions (Grizzetti et al., 2013). The 50-year data from 124 countries suggest that increased N fertilization involved low agronomical benefits and higher environmental risks. Different management practices have resulted in reduced NUE. Basically, the selection of crops or varieties with poor N uptake and assimilation followed by inefficient utilization through reduced N remobilization resulted in a lower N use efficiency (Dong and Lin, 2020). Furthermore, it is responsible for the loss of N from the soil and plant residue after harvesting the economic part (Kant et al., 2011). Galloway et al. (2003) reported that extensive crop cultivation over grasslands exposes the protected and stored soil organic carbon pool. Thus, it increases nitrate leaching and NH_3_ or NO_2_ and N_2_O emission, leading to environmental pollution. In South America, Africa, and Asia, reduced NUE was reported in areas devoid of cropping systems with biological N fixation such as soybean, beans, and groundnut (Herridge and Peoples, 1990; Liu et al., 2010). Similarly, intensive cropping without integration of livestock systems also reduced the N use efficiency at the local and global levels (Lassaletta et al., 2014). The promotion of synthetic N fertilizers rather than symbiotic N fixation resulted in poor N use efficiency (Lassaletta et al., 2014). Likewise, uncontrolled flood irrigation resulted in NO_3_^−^ leaching due to a negative charge and high solubility; furthermore, it creates anoxic conditions which lead to the development of denitrifying microorganisms (Chattha et al., 2022; Shabbir et al., 2022).

Environmental factors, mainly higher temperature and wind speed, increase the risk of NH_3_ volatilization (Chattha et al., 2022). It was found that an increase in soil temperature due to climate change increases the nitrification rate resulting in N loss and poor NUE (Engel et al., 2011). Higher soil compactness and wet conditions promote the denitrification process, whereas no-till and coarse soils showed higher leaching or volatilization/loss of nitrogen. In coarse soils, NH_4_NO_3_ fertilizer is subject to severe leaching and denitrification losses (Chattha et al., 2022).

**Figure 3:**
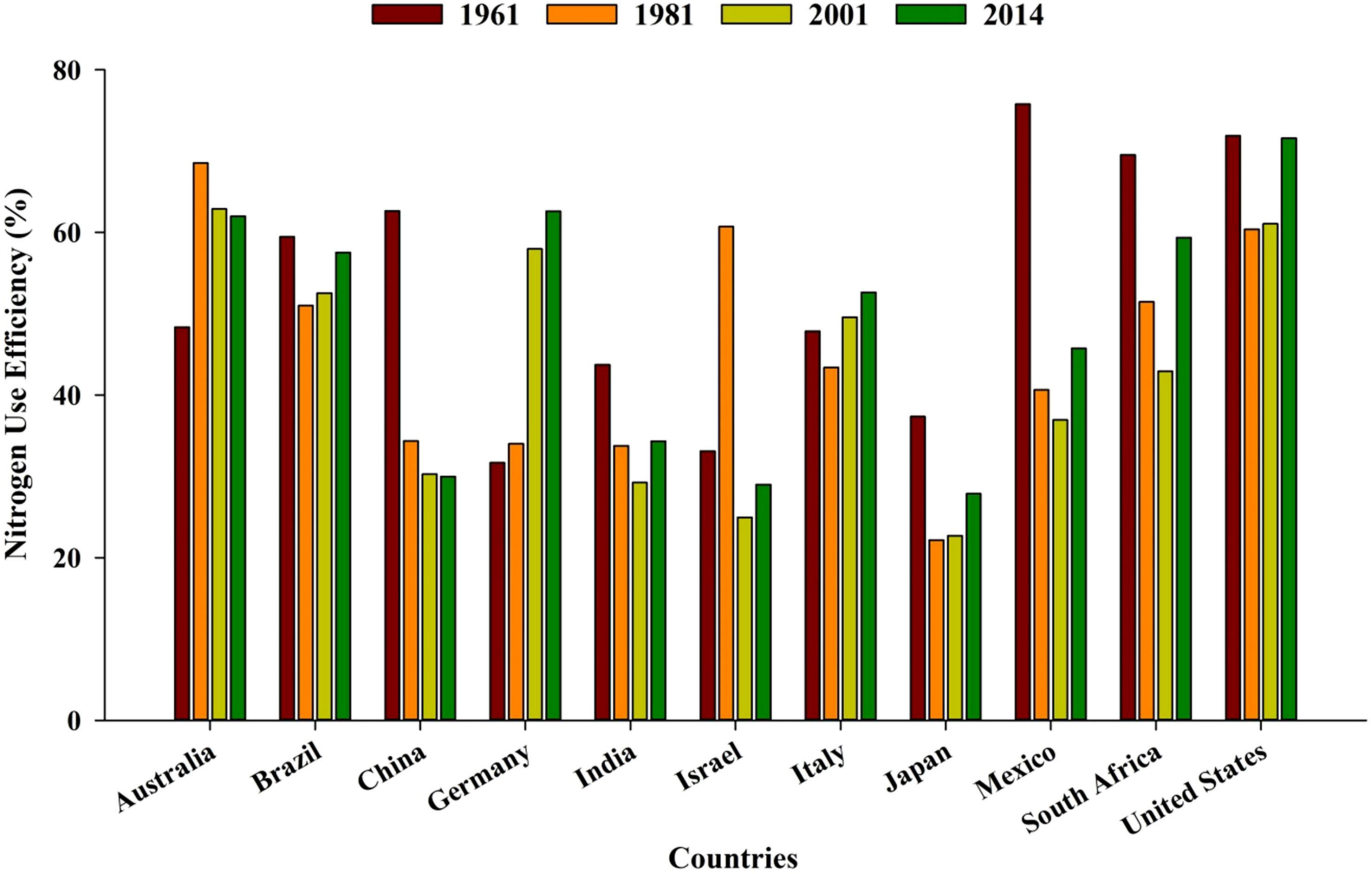
Average nitrogen use efficiency in different countries over the years (source: Lassaletta et al., 2014). https://ourworldindata.org/fertilizers http://creativecommons.org/licenses/by/4.0/deed.en_US.

Globally, the majority of countries are facing a decreasing trend of NUE (from 68% to 47%) over a period of five to six decades (1960–1970) (Lassaletta et al., 2014). Greater NUE in the initial years was probably due to higher native soil fertility, less use of additional nutrients, and favorable soil conditions (physical, chemical, and biological) (**Figure 3**). During the last decade, intensive management practices, monoculture, and increased use of off-farm input resources have resulted in low NUE (Lassaletta et al., 2014).

## 5 Consequences of poor NUE

Modern agriculture is entirely dependent on excessive N fertilizer application leading to ecosystem degradation and environmental pollution (Brender et al., 2013). According to estimates, 70% of applied nitrogen fertilizer is lost in the biosphere and affects the local and global atmospheric chemistry (Suthar et al., 2009). Nitrate pollution of groundwater in particular has led to numerous socioeconomic and environmental issues (Suthar et al., 2009). Nitrate contamination of drinking water is a major concern, particularly for children (Suthar et al., 2009; Brender et al., 2013). Continued consumption of NO_3_-contaminated drinking water (recommended limit of 10 mg NO_3_-N L^−1^) results in methemoglobinemia in children and gastric cancer among adults (Taneja et al., 2017). Moreover, NO_3_ or NH_4_^+^ contamination of water bodies promotes the growth of algae and other aquatic plants, which lowers the water’s oxygen level (Wild et al., 2001).

The oxide forms of N are highly reactive and harmful to the environment in many ways (Liu et al., 2019a). Excessive emissions of nitrous oxide and nitric oxide contribute to the formation of nitric acid, which is the key component of acid rain (Liu et al., 2019a). It significantly affects soil microbial communities and damages infrastructure (Liu et al., 2019a). Moreover, the atmospheric pollutant ozone is created when nitrous oxide combines with volatile organic pollutants (Karlsson et al., 2017). In this way, the loss of N leads to serious health and environmental problems. To avoid these consequences, the NUE of crops needs to be improved on a global basis.

## 6 Management and breeding approaches to improve NUE

### 6.1 Agronomic measures to enhance N use efficiency

#### 6.1.1 Conservation tillage system and NUE

The level of soil disturbance induced by different tillage practices affects soil N dynamics and plant N availability (Power and Peterson, 1998). For example, Francis and Knight (1993) reported that compared with conventional tillage systems, conservation tillage techniques reduced nitrogen availability. The absence of soil disturbance under the conservation tillage system can reduce the N mineralization rate, thereby decreasing the N availability to crops as well as the loss of N. In the conventional tillage system, however, increased oxidation of soil organic matter due to disturbance and exposure, as well as increased soil erosion, hastens the loss of soil organic matter (Schillinger et al., 1999). Soil organic matter loss caused by conventional tillage systems results in poor soil quality and low N availability. Therefore, the role of the tillage system will be vital for improving NUE.

The relationship between the conservation tillage system and NUE varies between studies, but overall NUE is often improved by the conservation tillage system (McConkey et al., 2002; Giacomini et al., 2010). Long-term conservation tillage systems (10–15 years) enhance the quantity of soil organic matter and increase the concentration of mineralizable organic nutrients at the soil surface layer (Sirivedhin and Gray, 2006), thereby improving the nutrient-supplying capacity of the soil (Van Den Bossche et al., 2009). As a result, conservation tillage systems that retain crop residues often result in higher crop yields and NUE compared with conventional tillage systems with a similar N application level (Stahl et al., 2019).

A long-term (10-year) study conducted in the southern United States of America showed that with the optimum application of N, cotton yields were higher in conservation tillage than in conventional tillage plots (Boquet et al., 2004). However, without N fertilizer application, the yields were lower in the conservation tillage system as a result of slow mineralization and immobilization of soil N (Boquet et al., 2004). For instance, in a study conducted in Kentucky, Phillips et al. (1980) found that fertilizer N applied on well-drained soil in a no-tillage system produced a greater (13.2 kg greater) corn yield per kilogram of applied N than under conventional tillage, but without N fertilizer, the corn yield was lower under a no-tillage system. On the contrary, crop residue retention, wetter soil surface, and anaerobic environments in no-till systems promote N immobilization, NH_4_ volatilization, and denitrification, negatively affecting N availability and NUE. In a wheat–fallow cropping system under the conventional tillage system, the N uptake was greater than that of stubble mulch systems. This is probably due to increased N immobilization in the stubble mulch system (Rasmussen and Rohde, 1991). Therefore, changes in N management, rate of application, and type of N fertilizer can improve NUE under conservation tillage systems. Overall, the role of conservation tillage and NUE requires more research to find practical compatibility.

#### 6.1.2 Managing N inputs for NUE

It has been demonstrated that NUE could be improved through management practices such as timing, rate, source, and placement of fertilizer application. These practices are considered fundamentals to N management and may be refined or supplemented by emerging and future technologies, but not replaced.

##### 6.1.2.1 Source

The chemical composition of N fertilizers influences the NUE of crops. Urea-based N sources can be lost through volatilization when hydrolyzed to ammonia (Eq. 16) and the effect is intensified when urea is surface applied (Chien et al., 2009). Slow-release N fertilizers have the potential to minimize N leaching and denitrification losses and to improve the synchronization of N release and uptake in accordance with crop demand (Shapiro et al., 2016). Similarly, coated N sources such as neem-coated urea, sulfur-coated urea, and slow-release synthetic urea-based fertilizers such as isobutylidene diurea (IBDU) and crotobylidene diurea (CDU) have also improved the NUE. Polymer-coated urea was also found to reduce N volatilization loss (23%–62%) and ammonia emissions (51.3%–91.3%) and improve NUE (3%–34%). The combined application of 150 kg N through urea + 2,000 kg manure and 90 kg N + 2,000 kg manure under normal and dry years, respectively, has recorded maximum grain yield and NUE by improving the nitrogen nutritive index and nitrogen productivity of wheat in dry land area (Liu et al., 2023). Furthermore, the combined application of N fertilizer (276 kg ha^−1^) with biochar (15 t ha^−1^) produced the maximum yield of maize and the NUE (46.3%). Zhang et al. (2023) demonstrated that integrated application of 180 kg N ha^−1^ + 900 g Se ha^−1^ utilized the maximum resources and recorded maximum apparent recovery efficiency of N, agronomic N use efficiency, partial factor productivity, NUE, and grain yield of wheat.

##### 6.1.2.2 Rate

Before determining the amount of fertilizer to apply, consider the soil’s nutrient-supplying capacity through a regionally appropriate soil testing program. Excessive fertilizer application leads to losses from the system, environmental problems, and economic losses to farmers. On the other hand, an insufficient nutrient application can exhaust soil fertility and lead to nutrient mining (degradation) and poor long-term soil productivity. Optimizing the nutrients’ rate based on soil status and crop requirement is the right way to improve NUE.

Typically, N fertilizers are applied either in single or two split applications. Split application of N at various crop stages is effective at increasing NUE. The application of 120 kg N ha^−1^ proved optimal to produce a higher grain yield and NUE in direct-seeded rice than 60 and 180 kg N ha^−1^ (Mahajan et al., 2012). A higher fodder maize seed yield (3.80 t ha^−1^) and N utilization were recorded for 120 kg N ha^−1^ in a semiarid region (Halli et al., 2019). Application of 180 kg ha^−1^ has been recommended to achieve higher grain yield, NUE, and protein yield of buckwheat (Wan et al., 2023). Hu et al. (2023) reported that among the rates of N studied (0, 150, 200, 250, and 300 kg ha^−1^), fertilization at the rate of 250 kg N ha^−1^ recorded a maximum grain yield, maximum grain N accumulation, improved aboveground dry biomass and N metabolism enzymes, and increased NUE in corn.

Site-specific N scheduling could be an alternative option to the blanket application of N. Results from a study conducted in 107 farmers’ fields indicated that the leaf color chart (LCC)-guided N management in hybrid rice had decreased N requirement by 25% without compromising the crop yield (Bhatia et al., 2012). Therefore, LCC can be further explored as a diagnostic tool to help farmers make appropriate decisions about N fertilizer applications throughout the crop cycle. However, the use of sensor-based N application techniques is still at a nascent stage in many parts of the world.

##### 6.1.2.3 Placement

Fertilizer placement nearer to the root zone of crop plants, as opposed to even distribution in the field, has the potential to minimize N losses. The incorporation of fertilizers in the soil (*via* tillage or injection) is recommended over broadcasting (Ladha et al., 2005). The placement of N fertilizer under the seeds at the time of planting, band application, and fertilizer injection increased the NUE and reduced the NH_3_ volatilization compared with broadcasting in winter wheat (Dao, 1998; Ladha et al., 2005).

Deep placement of the USG fertilizer resulted in better N recovery efficiency (49%) compared with the broadcasting method (37%) in Australia (Schmidt et al., 2002). Granular ammonium nitrate fertilization at the depth of 20 cm below the soil surface recorded the highest NUE (134%), N recovery efficiency (18.1%), and grain yield (11%) in spring wheat and barley (Rychel et al, 2020). Qiang et al. (2022) noted that placement of controlled release urea at the depth of 16 cm achieved maximum grain yield, water productivity, partial factor productivity, and NUE in rainfed spring maize in Northern China.

Fertigation, or co-application of N with irrigation, is a viable option for the improvement of NUE. N fertilization at 15 cm depth increased grain yield (13.9%–98.9%), NUE (7.1%–44.3%), and N absorption (6.5%–38.0%) in summer maize (Chen et al., 2023). This approach gives the farmer with the proper equipment the flexibility to engage in multiple applications of low rates to minimize exposure to losses and optimize the opportunity of the crop to take up the right amount at the right time.

##### 6.1.2.4 Timing

The timing of fertilizer application should coincide as close as possible with crop nutrient demand to avoid nutrient loss. For instance, in single applications, part of the applied nutrient is absorbed by plants, while a substantial portion is vulnerable to loss. Improved N partial factor productivity, agronomic N efficiency, N recovery efficiency, physiological efficiency, grain yield, and N uptake may be optimized when N is applied in four splits at the sowing, 6th leaf stage, 12th leaf stage, and silking stage in maize (Zhou et al., 2019). However, commercial-scale agriculture will likely avoid multiple trips across the field and traffic when the crop canopy has closed to reduce fuel, compaction, and crop damage. Likewise, the application of N fertilizer in three splits has increased the wheat grain yield and N recovery use efficiency (Liu et al., 2019b).

Ranatunga et al, 2018 indicated that more than 6 t ha^−1^ grain yields can be achieved in dry direct-seeded rice production systems when urea application is delayed by 10 days after sowing or split application compared with the blanket application. Although optimized in this way, the commercial-scale application on flooded rice will be impossible without aerial application. Split application of N at the time of sowing and later stages (V12, R1, and R2) increased the plant uptake, photosynthetic efficiency, and grain yield and improved NUE in summer maize (Deng et al., 2023). Late and split application of N during jointing, booting, anthesis, and grain filling stages through microsprinkler irrigation increased grain yield, protein concentration, and NUE of wheat by 5.8%, 8.6%, and 15.8%, respectively, as compared with the conventional method of fertilization and irrigation (Yao et al., 2023). N application with basal to top dressing ratio of 2:8 between the sowing and jointing stages recorded maximum dry matter yield, crude protein, N recovery, water, and N use efficiency of forage maize in a semiarid region of China (Ma et al., 2023). Hence, the split application of N would be superior to the blanket application, though the number and timing of these applications will be limited due to the practical considerations mentioned above.

#### 6.1.3 Cropping system and NUE

Nitrogen use efficiency is also dependent on the ability of the cropping system (Ortiz-Monasterio et al., 1997; Reddy, 2011). Crop diversification can improve soil structure, soil health, vertical nutrient stratification, and mycorrhizal fungal interactions, as well as offer diversity in crop residues. A potential cropping system could help improve N availability and plant uptake (Tisdall and Oades, 1979; Lehman et al., 2012). Cereal- and legume-based cropping is the best system for leaving more residual N accumulation (Lehman et al., 2012). In a study with fallow followed by rice and legume followed by rice systems in Japan, the fertilizer NUE was higher for the legume (broad bean) followed by rice with 40 kg N application compared with fallow followed by rice in a clay loam soil (Rahman et al., 2009). Similarly, in a 20-year study on clay loam soil in Ontario, Canada, Gaudin et al. (2015) reported an increase in maize fertilizer NUE when winter wheat is inserted into a maize–soybean (especially when wheat is under-seeded with red clover) cropping system. In another study conducted in China, Li et al, 2022 found a higher N uptake and N harvest index in faba bean when intercropped with wheat compared with sole faba bean. The benefits associated with crop rotation and intercropping are mainly due to the facilitation through interaction between legumes and cereals and shallow-rooted and deep-rooted crops. Therefore, the rotation of crops with different depths of roots can improve soil structure and stability (Obalum and Obi, 2010) and enhance resource use efficiency (Halli et al, 2019). Tap-rooted crops can more easily penetrate compacted soil layers than shallow or fibrous-rooted crops, which serve to enhance the water and N use efficiency of the overall system (Chen and Ray, 2010). In Denmark, van Oosterom et al, 2010 observed a maximum mineralized N (81 kg N ha^−1^) within the rooting zone of pea–cabbage compared with the onion–cauliflower cropping sequence, where the mineralized N was only 52 kg ha^−1^ within the root zone. The selection of varieties/crops with different root systems, varied capacity to fix atmospheric N, and higher biomass production is an effective strategy to enhance NUE that deserves future research attention.

#### 6.1.4 Inclusion of cover crops and forage crops in the cropping system

Nitrogen use efficiency of plants depends on the rate of soil N used by roots and accumulation in different plant parts such as the stem, leaf, and harvestable portions. Therefore, NUE is influenced by the inclusion of cover crops in a cropping system. The inclusion of high biomass-producing crops such as cover crops and dual-use forage crops can enhance the overall NUE of any system (Reicosky and Forcella, 1998). Cover crops are the crops planted in the off-season when the land is otherwise left uncultivated. Leaving land fallow increases the likelihood of soil erosion and nutrient leaching. Cover crops can help to protect the soil from loss, keep living roots in the soil as much of the year as possible, and recycle nutrients.

Cover crops with low C:N ratio residues (legume) can hasten the mineralization of organic N which may be responsible for the high NUE of the main crops (Franzluebbers et al., 2014). However, the cover crops with high biomass and high C:N ratio residues can lead to the immobilization of N, decreasing NUE for the following cash crop. A simulation model study using NLEAP (N Leaching and Economic Analysis Package) predicted that the inclusion of winter cover crops increased the NUE of lettuce by 3.1 kg per 4.5 kg of available N (Delgado, 1998). The cover crops in this study included winter wheat and rye, which were modeled to recover and retain soil NO_3_-N in tissue, preventing leaching loss and fertilizing the next crops. Similarly, planting cereal rye crops after no-till corn has reduced N leaching by 100% (McCracken, 1989). The CERES-N model modified by Quemada and Cabrera (1995) includes important considerations outside of simple C:N ratios to predict the mineralization or immobilization potential of cover crop residues. The model requires inputs for water-soluble carbohydrates, cellulose/hemicellulose, lignin, total C, total N, and C:N ratio.

Forage crops (often perennials) also contribute to the reduction in N loss and improved NUE. For example, a study from the USA reported that a perennial, such as alfalfa, reduced NO_3_-N leaching by 10-fold over a corn–soybean rotation or continuous corn systems (Randall and David, 2001). Moreover, persistent roots of forage grasses are important to bind the soil particles together to develop a stable soil structure and potentially capture N from 1.5 m deep in the soil. Thus, surface available N can be utilized by subsequent crops to improve NUE.

### 6.2 Genetic resources and breeding approaches to enhance/improve NUE

During the green revolution and post-green revolution, high fertilizer-responsive cultivars have been favored owing to low N-fertilizer costs. Though there are contradictory reports that under low N, more N-responsive modern varieties still perform better than historical varieties (Ding et al., 2005; Echarte et al., 2008), breeding efforts to develop high fertilizer-responsive cultivars under high fertilizer conditions have resulted in high-yielding cultivars with poor NUE (Garnett et al., 2015). As a consequence, yielding increases are fast approaching a theoretical limit with given physiological and genetic potential of crop cultivars under high N availability (Ali et al., 2018). To narrow down the demand–supply gap of food amid decreasing farmland and depleting soils around the globe without further magnifying environmental impacts, breeding strategies to improve the NUE of crop cultivars are becoming the prime focus of agricultural researchers (Fiaz et al., 2021; Ciampitti et al., 2022). Breeding for high input-responsive cultivars, occurring during the last five to six decades, is different from breeding for NUE. For NUE, the inherent capacity of the plant has to be improved and selected to facilitate efficient uptake and to use N and produce higher yield under moderate or marginal N availability (Anbessa and Juskiw, 2012). Therefore, breeding for high NUE is mainly aimed at realizing maximum benefit by reducing the N application rate while maintaining the high yield level.

#### 6.2.1 Breeding approaches to improve NUE

Although there has been a consensus on the need to increase the NUE of crop plants through breeding, practically, no breeding program is primarily dedicated worldwide for this purpose, to the best of our knowledge. Theoretically, there may be different ways to improve NUE through breeding, such as overall consideration of grain yield or biomass growth under limited N conditions, selection and improvement of specific traits that contribute to high NUE, or introduction of the foreign gene. However, indirect selection for yield has been the common method for achieving higher NUE (Cormier et al., 2016).

NUE is considered a complex trait. Modifications in traits such as plant height, tiller number, dry weight of shoots and roots, grain yield, spikelet number, number of filled grains per panicle, 1,000-grain weight, and chloroplasts were reported to improve NUE (Lawlor, 2002; Zhao et al., 2011a; Hamaoka et al., 2013). Breeding targets for genetic improvement of the plant may be grouped into two major categories: first, improving N uptake efficiency by increasing uptake capacity (Le Gouis et al., 2000) and breeding for ideal root morphology (Liao et al., 2006) and, second, improving N utilization efficiency by modifying the leaf area index, specific leaf N, and biomass yield (Gastal and Lemaire, 2002) and by delaying the senescence (Foulkes et al., 2009).

#### 6.2.2 Improving N uptake efficiency

Before initiating the new breeding efforts to create genetic variability for high NUE in modern crop cultivars, the rich genetic resources conserved in different gene/seed banks of the world should be explored for screening high NUE lines. There is proven genetic diversity for root N uptake in plants (Pereira et al, 2010; Le Gouis et al., 2000), and exploiting this property requires researchers to understand the underlying mechanism of higher root uptake.

Root morphology plays a critical role in modulating N uptake by plants (Garnett and Rebetzke, 2013). Plants with rapid root growth can minimize N losses that occur through various field processes (Gastal and Lemaire, 2002). Anbessa and Juskiw (2012) observed that barley plants with higher root dry weight and volume assessed at the five-leaf stage showed higher NUE than normal plants. Improvements in root traits such as length of root, root-length density, the radius of the root, root surface area, and number, length, and density of root hairs (Wang et al., 2006) are associated with greater N uptake in plants. Breeding efforts for enhancing root-related traits are essential for improving NUE. However, the limited scope of large-scale and high-throughput root phenotyping creates obstacles in breeding programs for selecting and screening specifically for such beneficial root architecture (Fiorani and Schurr, 2013).

#### 6.2.3 Improving N utilization efficiency

The uptake of additional N must match with the metabolism of the plants to avoid systemic feedback control of metabolites representative of the whole-plant N status (Nacry et al., 2013). The uptake and utilization of N for the entire plant growth period can be separated into two phases: pre-anthesis and post-anthesis (Cormier et al., 2016). At the pre-anthesis stage, plants take up N, and the whole-plant system utilizes it upon receiving fractional interception of light at the start of the stem elongation phase. However, at post-anthesis, once grains appear, plants begin partitioning available N for higher grain yield, jeopardizing the simultaneous improvement in grain yield and protein content (Oury and Godin, 2007). Higher N utilization is possible under low N supply through an increased specific leaf N area (SLN), which is reported to be associated with the embryo size of the plant (López-Castañeda et al., 1996) and earlier canopy closure (Rebetzke and Richards, 1999). Physiological conditions wherein N is more efficiently utilized are associated with the abundance of prostrate leaves during vegetative growth and semi-erect to erect leaves during later vegetative and reproductive stages. This can be difficult for plant architecture to manipulate (Cormier et al., 2016). Normally, at the post-anthesis stage, the grains draw N from the stem and rachis in cereals and then from leaves if necessary. However, the stay-green plant types are prone to supply N to growing grains slowly and thus impact the balance in the N demand–supply framework (van Oosterom et al., 2010). Researchers are in consensus that physiologically important traits that directly or indirectly improve N utilization are taken into consideration in breeding programs, in addition to the common target traits. However, assessing those traits on the bulk scale is a question of technological advancement, resources available to the breeders, and practical limitations (Cormier et al., 2016).

### 6.3 Biotechnological approaches to enhance NUE in crops

The integration of molecular tools, such as genomics and marker-assisted breeding, into traditional breeding programs has revolutionized genetic enhancements for various intricate traits in crops (Jagannadham et al., 2019). The incorporation of these tools has significantly increased the efficiency of the selection process, resulting in a reduction in the time and resources required to develop improved varieties or hybrids. Recent advances in genomics have further accelerated the generation of genomic resources for many crops, providing breeders with more data and insights into the genetic makeup of crops, ultimately leading to more effective breeding strategies (Kumar et al., 2018c; Jagannadham et al., 2019). Ultimately, these resources can be exploited for identifying, characterizing, and developing molecular markers linked to N-responsive genes in crop plants (Yang et al., 2017; Lenka et al., 2018). Two molecular approaches can be explored for improving NUE in crops; one is through a traditional breeding strategy combined with genomic selection, and the other is a transgenic approach, which would target specific NUE-associated genes for the genetic engineering of the plant (Good and Beatty, 2011; McAllister et al., 2012; Kumar et al., 2018c).

#### 6.3.1 Genes/QTLs associated with NUE

It is of utmost importance to identify genes or QTLs that govern NUE to enable the breeding of crops with high NUE using approaches such as marker-assisted selection (MAS) and genomic selection. Nutrient use efficiency is a complex trait, and as a result, several research groups have undertaken efforts to map the genetic loci in correlation with specific traits (Balyan et al., 2016; Kumar et al., 2018b; Mălinaş et al., 2022). In rice, 20 single QTLs (S-QTLs) and 58 pairs of epistatic loci (E-QTLs) were identified for the grain N, straw N, shoot N, harvest index, grain yield, straw yield, and PE in low N and ordinary N conditions. Harvest index and grain yield were positively correlated with PE in both conditions (Cho et al., 2007). In another study carried out with rice, four QTL clusters harboring QTLs for both NDT and NUE traits were identified (Wei et al., 2012). In European winter wheat, a genome-wide association study using 214 varieties identified 333 genomic regions associated with 28 traits related to NUE (Cormier et al., 2014). For the second approach, specific NUE-associated genes should be identified. Some of the efforts successfully mapped genes and identified QTLs. In maize, a meta-analysis of published NUE QTLs revealed 37 “consensus” QTLs, of which 18 were detected under low N conditions. Comparing expressed sequence tags (ESTs) associated with low N stress response, N uptake and transport, and assimilation with the QTL map has resulted in identifying candidate NUE-associated genes. Among those genes, nine candidates introgressed into Ye478 have significantly altered grain yield/yield components (Liu et al., 2012). Five significant QTL clusters associated with large-rooted architecture and high N uptake efficiency (NupE) were identified in maize. The root system architecture (RSA), such as that found in maize, has an essential role in N acquisition. NupE had significant phenotypic correlations with RSA (Li et al., 2015). Three QTLs, NUE1a, NUE1b, and NUE2, were identified in maize for NUE (Mandolino et al., 2018). Under N starvation, the expression of *TaNLP7* displayed enhanced expression in root and shoot tissues of the high NUE genotype (Kumar et al., 2018a). Forty-seven genes are known to involve N uptake, metabolism, and distribution in maize (Wani et al., 2021). In barley, 10 independent mapping studies were screened and a number of NUE-associated genes that control complex physiological traits were mapped (Han et al., 2016). Even though a large number of reports claim to be identifying QTLs for NUE, some of them are yet to be validated. Since NUE involves a myriad of factors, the traditional breeding strategy combined with MAS will be cumbersome. Therefore, exploiting genomic selection for improving NUE will speed up the development of superior genotypes by combining high-throughput phenotyping and genotyping (Han et al., 2016; Kumar et al., 2018c; Stahl et al., 2019). In wheat, four QTLs, viz., *QNue.151-1D*, *QNue.151-4A*, *QNue.151-6A*, and *QNue*.*151-7D*, were associated with NUE; one QTL, *QNupe.151-4A*, was associated with N uptake efficiency; and one QTL, *QNute.151-4A*, was associated with N utilization efficiency (Brasier et al., 2020). The details of the QTLs identified in the crop plants are given in **Table 4**.

**Table 4:** QTLs identified in various crops related to NUE.

S. no.	Crop	QTL	Description	References
1	Wheat	*Qnue.151-6A*	Involved in the assimilation of ammonium into amino acids	Brasier et al. (2020)
2	Wheat	*QNue*.*151-1D*	Indicate a role in seedling vigor	Brasier et al. (2020)
3	Wheat	*QNue*.*52-7A*	Significantly increased NUE under the reduced N rate and resulted in higher NUE	Brasier et al. (2020)
4	Wheat	36 QTLs	13 QTLs for NUE, 13 QTLs for NUpE, and 6 QTLs for NUtE	Singh et al. (2022)
5	Rice	*QAE_2.1*, *qAE_4.1*, *qAE_6.1*, and *qAE_12.1*	Agronomic efficiency of applied nitrogen in terms of P conditions	Jewel et al. (2019)
6	Wheat	*QSnc.2*, *Qtnc*, and *QRsnc.1*	Three N uptake efficiency (NUpE) (GNC, StNC, and ANC)	Zhang et al. (2019)
7	Wheat	*Qsnue.2*, *QTnue.4, Qgnue*, and *QAnue.3*	Three N utilization efficiency (NUtE) traits (GNUE, StNUE, and ANUE)	Zhang et al. (2019)
8.	Potato	*NUE_D_LN1*, *NUE_K_HN*, *NUE_D_LN2* and *NUE_K_LN*	Nitrogen use efficiency (NUE)	Getahun et al. (2020)
9	Maize	*NUE1a*, *NUE1b*, *NUE2*	N use efficiency for grain production	Mandolino et al. (2018)

#### 6.3.2 The role of small RNAs and transcription factors in the regulation of nutrient response

**Figure 4:**
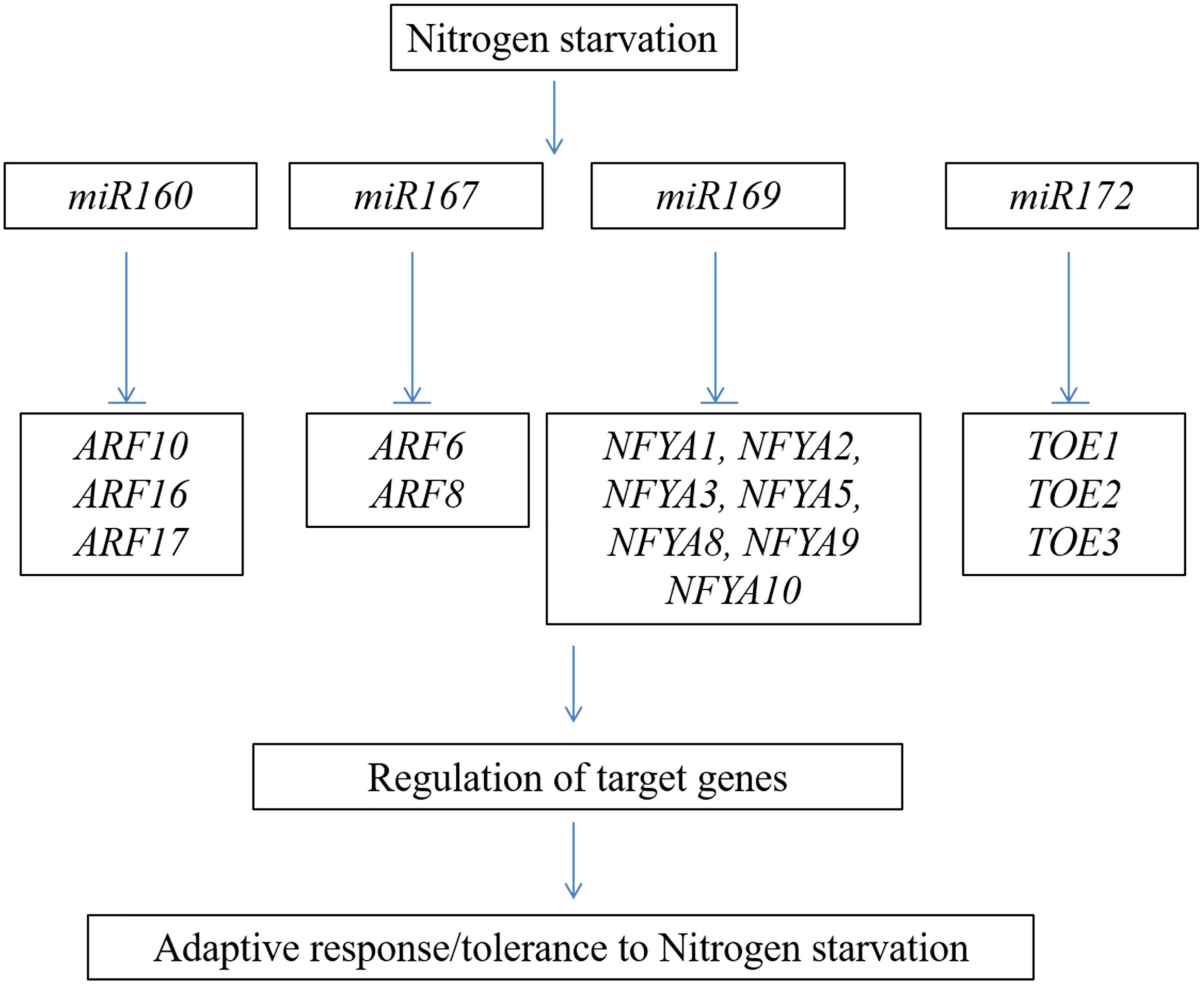
Schematic representation of important nitrogen-responsive *miRNA*s and their transcription factor targets involved in nitrogen starvation response.

The role of small RNAs in regulating the nutrition assimilation/starvation response is well documented in many crops (Balyan et al., 2016). A total of 126 long non-coding RNAs (lncRNAs) were altered during N starvation, and these RNAs regulate various protein-coding genes involved in diverse cellular functions (Chen et al., 2016). Forty-four *miRNAs* are differentially regulated under high and low N conditions (Li et al., 2016). Most of these targets were found to be the genes encoding for the transcription factors. The important miRNAs and transcription factors involved in the N starvation response in *Arabidopsis* are shown in **Figure 4**. In *Arabidopsis* and maize, the expression of *miR167* was enhanced under N starvation conditions (Xu et al., 2011; Balyan et al., 2016). *miR167* regulates the lateral root growth response to N starvation in *Arabidopsis* (Gifford et al., 2008). Conversely, downregulation of the transcription factors *ARF10*, *ARF16*, and *ARF17* by N-responsive *miR160* regulates the process of seed germination and development of the seedling after post-germination under N-deficient conditions (Liu et al., 2007; Hao et al., 2022). Downregulation of *miR169* enhances the expression of the *NFYA* transcription factors; these genes regulate the function of the nitrate transporter genes, viz., *AtNRT1.1* and *AtNRT2.1* (Zhao et al., 2011b). These studies showed the involvement of small RNAs and their functional importance in inducing/repressing multiple genes in response to N assimilation/deprivation and regulation of root development in plants. In wheat, simple sequence repeat markers developed from *miR171a* effectively group the panel of wheat genotypes into N-efficient and non-efficient markers. These markers can be employed to characterize the wheat germplasm/breeding lines in crop breeding programs (Sagwal et al., 2022).

#### 6.3.3 Genetic engineering

Plants have evolved mechanisms to alter the molecular machinery in response to N availability (Gaudinier et al., 2018). Yang et al. (2017) identified 1,158 and 492 genes that were differentially expressed in leaf sheaths and roots, respectively, after 12 h of N starvation in rice. Conversely, in *Dunaliella salina*, 3,127 were differentially expressed (2,380 genes were upregulated and 747 were downregulated) under N starvation (Lv et al., 2019). In maize, *ZmGLK5*, *bZIP108*, *CLC-a*, and *miRNA399b* genes play a significant role in regulating genes in response to N (Jiang et al., 2018). The NIGT1/HRS1s transcriptional repressors are essential in regulating N starvation response during high N availability (Kiba et al., 2018). The CLE peptides and the CBL7 and TAR2 proteins regulate root architecture in response to N starvation (Kiba et al., 2018); the DUR3 and AMT family proteins play an important role in the uptake of urea and ammonium, respectively, during N starvation (Krapp et al., 2014). The nitrate transporters, NRT1/NPF and NRT2, regulate nitrate uptake (Krapp et al., 2014).

The availability of high-throughput genomics tools and efficient transformation systems in model crops further eases the functional validation of NUE (Muthusamy et al., 2016; Lenka et al., 2019). Several attempts have been made to develop transgenics with high NUE. Overexpression of *AtDof1*, *AtGS1*, and *AtGS2* enhances the N assimilation in transgenic tobacco lines grown under N-starved conditions compared with wild-type plants (Wang et al., 2013). Transgenic overexpression of *OsDof25* modulates C and N metabolism in transgenic *Arabidopsis* lines during an increased supply of N (Santos et al., 2012). Plant species comprising the C_4_ photosynthetic pathway have evolved highly efficient molecular mechanisms of carbon fixation. C_4_ plants exhibit high radiational, N, and water use efficiencies compared with species with the C_3_ photosynthetic mechanism (Ghannoum et al., 2011; Muthusamy et al., 2019). Engineering the genes involved in the C_4_ photosynthetic pathway in C_3_ plants remains an essential strategy for enhancing the NUE in C_3_ crops (Lin et al., 2019; Muthusamy et al., 2019). Moreover, the availability of N regulates the ethylene and jasmonic acid hormone signaling, thereby regulating the plant response to pathogen infection (Vega et al., 2015; Farjad et al., 2018). *miRNA*s are known to play an important role in regulating the function of N-responsive genes during N-limiting conditions (Nguyen et al., 2015; Zuluaga et al., 2017). Thus, the identification of gene regulatory networks, including small RNAs involved in regulating the stress response, will further help to understand the development of stress-responsive crops with high NUE (Muthusamy et al., 2017; Zuluaga et al., 2017; Farjad et al., 2018). The details of the QTLs identified in the crop plants are given in **Table 5**.

**Table 5:** Genes identified in various crops related to NUE.

S. no.	Crop	Gene	Description	References
1	Rice	*OsNRT2.1B*	Involved in nitrogen uptake and utilization	Naz et al. (2019)
2.	Eggplant	*WRKY33*	Involved in eliciting several genes associated with low N response	Mauceri et al. (2021)
3.	Maize	*ZmAMT1;1a*	Enhances plant tolerance to low ammonium	Zhao et al. (2018)
4.	Wheat	*TaNRT2.1-6B*	Improves N uptake from the soil under both limited and sufficient N conditions	Li et al. (2022)
5.	Rice	*NIGT1*	Regulates the expression of nitrate-inducible genes in a feedback loop	Ueda et al. (2020)
6.	Rice	*OSA1*	Involved in ammonium absorption and C fixation	Zhang et al. (2021)
7.	Rice	*OsNAC42*	Regulates the transcription of a nitrate transporter that confers high nitrogen use efficiency	Tang et al. (2019)
8.	Maize	*THP9*	Increases nitrogen use efficiency	Huang et al. (2022)

## 7 Conclusion

In global agriculture, the low-efficiency uptake by crops of applied N fertilizer is a major concern because of its negative impact on production costs and the environment. To improve NUE in crops, modern agronomic, breeding, and biotechnological strategies should be incorporated to supplement fundamental nutrient management. Agronomic practices such as precise timing and placement of N fertilizer, site-specific nutrient management, conservation tillage, crop residue retention, and cultivation of high biomass crops can enhance NUE under various soil and climatic conditions. NUE is a multifaceted trait that involves physiological, biochemical, and molecular regulations. Therefore, the engineering of N-responsive genes through genome editing has great potential for improving NUE in crops. To breed superior genotypes with high NUE, the use of genomic selection combined with speed breeding techniques in breeding programs is expected to be a valuable approach in the future.

## Author contributions

PG, SM, MB, RV, PJ, AM, HH, RR, SB, VP, GT and ML: manuscript writing and editing. All authors contributed to the article and approved the submitted version.
